# Habit Representation Based on Activity Recognition

**DOI:** 10.3390/s20071928

**Published:** 2020-03-30

**Authors:** Jaeryoung Lee, Nicholas Melo

**Affiliations:** Department of Robotic Science and Technology, College of Engineering, Chubu University, Kasugai, Aichi 487-8501, Japan; nicholas.bastos@gmail.com

**Keywords:** distributed sensors, activity recognition system, habit assessment, elderly support

## Abstract

With the increasing elderly population, attention has been drawn to the development of applications for habit assessment using activity data from smart environments that can be implemented in care facilities. In this paper, we introduce a novel habit assessment method based on information of human activities. First, a recognition system tracks the user’s activities of daily living by collecting data from multiple object sensors and ambient sensors that are distributed within the environment. Based on this information, the activities of daily living are expressed using Fourier series representation. The durations and sequence of the activities are represented by the phases and amplitudes of the harmonics. In this manner, each sequence is represented in a form that we refer to as a behavioral spectrum. After that, signals are clustered to find habits. We also calculate the variability, and by comparing the explained variance, the types of habits are found. For an evaluation, two datasets (young and elderly population) were used, and the results showed the potential habits of each group. The outcomes of this study can help improve and expand the applications of smart homes.

## 1. Introduction

With the rapid growth of the elderly population, several countries are preparing to face super-aged societies. Thirteen countries will become super-aged societies by 2020, and more are expected by 2030 [1]. According to the government of Japan, which is already a super-aged nation, resolving the “social isolation and solitary deaths of older persons under the collapse of local communities” is a crucial challenge [2]. To address this issue and reduce the burden on caregivers, systems have been implemented to monitor the activities of daily living (ADLs) for elderly people. Numerous studies have shown that elderly populations can be supported through a variety of applications such as robots and smart home systems [3]. However, the challenge of recognizing ADLs of the elderly population using a smart environment remains, and it is being resolved through focused studies. Aicha et al. identified that the presence of multiple persons in a same room increases detection difficulty [4]. In a later work, the same research group solved that limitation by equipping multiple sensors throughout several rooms of an apartment [5]. Chernbumroong et al. suggested a practical assisted living system which was designed with consideration for acceptance by elderly people and for their privacy issues using low-cost, low-battery consumption sensors [6]. Rantz et al. conducted a study with 25 elderly participants (average age of 87.99) that stayed in a sensor-equipped facility to test an alert system for a year, which is relatively long-term study in comparison to other studies. The system provided efficient monitoring of residents to notify clinicians or caregivers. These studies indicate that a realistic solution for sensory technology is needed and for researchers to be more focused on real-world applications, such as in assisted living systems. Thus, our study is focused on the development of a practical system using simple sensors to identify activity patterns through a recognition system.

### 1.1. Related Work

Various approaches to ADL recognition in smart environments have been developed, and researchers have begun focusing on detecting anomalous patterns of behavior in the daily routines of users. Huang et al. developed a smart system that could detect sudden changes in elderly people’s activities [7]. Meng et al. designed a habit model that could identify an activity and its time interval [8]. To understand the habits of users, ADL recognition should be performed first. Wang et al. categorized sensor types as body-worn sensors, object sensors, ambient sensors, and hybrid sensors [9]. Acceleration and angular velocity have commonly been extracted from body-worn sensor devices [10] or smartphones [11] by several researchers for activity detection. For more context information, the work in [12] used a body-worn sensor to acquire information such as body movement, temperature, and humidity, and applied supervised deep learning. Yang et al. [13] used a dataset including information acquired from body-worn sensors and ambient sensors. Hybrid sensors acquire multiple types of data, and were utilized in some works to obtain rich information on human behaviors [14]. Chavarriaga et al. used a recognition system equipped with body-worn, object, and ambient sensors to create a sensor-rich environment. This system provided rich information of human activities such as gestures and multi-modal locomotion [15]. Using a large number of sensors improves detection in complex scenarios where several people exist in the same space, such as family members or other care-givers [5], or where activities that are discontinuous or with a varied order are performed [16]. Such monitoring systems improve the detection of abnormal behavior based on patterns in user ADL data, such as forgetting activities and repeating them, or disruptions in sleep due to dehydration [17]; these systems could remind users of important activities, particularly for elderly people. Arifoglu and Bouchachia studied the abnormal behavior of elderly people with dementia by finding differences among their routine patterns [17]. Chernbumroong et al. proposed an activity recognition system for supporting elderly people using simple body-worn sensors rather than complex sensors, and the results showed a high classification rate [6].

### 1.2. Contributions and Paper Overview

Our proposed system uses PIR, pressure, RFID, and current sensors which are relatively simpler than the sensory system used in the studies mentioned in Section 1.1. There are several reasons for using object sensors and ambient sensors, which exclude visual information, rather than using body-worn sensors or cameras. First, the majority of elderly people want to avoid camera observations to protect their privacy, even though they accept the use of sensor technology in their residences [18]. Second, current sensors can be low-cost and small in size relative to more complex sensors, while still being capable of detecting the activities of elderly people [6]. Moreover, body-worn sensors could be an additional burden for elderly people. The purpose of this work is to develop a method to represent the habits of people through ADL data. In our previous work, we developed a human activity recognition system based on data obtained from distributed sensors [19]. The system did not use body-attached sensors or visual data, but it still achieved high recognition rates despite the limited information. The concept of the proposed method is to represent user habits in a numerical format based on the sequence and duration of the observed activities. This numerical representation is used to extract and analyze features regarding user activities and habits. Because the concept of habits can have a wide range of definitions, it is important to clearly define the ones used in this work.

The remainder of this paper is organized as the follows. Section 2 presents the structural overview of the habit assessment system. Section 3 explains the numerical representation of human activities and habits. Section 4 describes the data collection environment and the two datasets. Section 5 presents the results for each dataset. Finally, Section 6 discusses the unanticipated results and future work.

## 2. Approach towards Habits

Several studies have suggested a system or model for human activity recognition obtained from compact applications, which use small and inexpensive sensors with low power consumption, as stated by Chernbumroong [6]. These studies aimed to provide high recognition rates and particularly focused on the detection of abnormal behavior [20]. The sensing modalities varied depending on the objectives of system, and some of the studies used a combination of sensors to extract more information of the activities [9]. The ultimate purpose of these studies was to understand the ADLs of people autonomously and to assist them; for this, the system needs to assess the person’s habits as well. However, few studies have expanded or applied a human activity recognition system to habit assessment. Ordóñez et al. used Bayesian methods to identify the abnormal activities of users. The three features (sensor activation, sequence, and duration) considered in their work are similar to those in our proposed approach, but the model struggled to represent the habits of users [21]. The habit assessment system in this work mainly addresses two features, the sequences and duration, and it is capable of estimating the routine of a user’s daily living.

In several studies, the term “habit” referred to the routines of the daily lives of humans [8]. However, most studies focused on the detection of abnormal behavior based on individual activities. Anomalous habits in those studies mainly included sudden changes such as falls. The habits in this work indicate not only the activities, but also each sequence and series of sequences. Leotta et al. suggested the definition of a habit as a sequence of activities [22]. This overall definition includes the basic concept of a habit being a pattern observed from a start time to an end time for each particular activity. For example, if the user takes a nap from 12:00 (start time) to 13:00 (end time) almost daily, it can be identified as a habit. According to its basic definition, the habit assessment system classifies frequently observed sequences when the user performs a set of simple activities. Let us assume that the user always takes a nap for approximately 1 h after having lunch for 30 min. These two activities with two different durations are observed as a habit set. The main components can be repetitive or changeable, so we refer to it as a *dynamic habit*. Although an analysis of simple habits can aid in the detection of abnormal behavior, there is insufficient information regarding the frequency of activities performed as part of a simple habit. By contrast, a dynamic habit can provide more information regarding the user and will help build a more efficient and effective support system.

### 2.1. Activity Recognition System

In our previous work [19,23,24], we have developed an activity recognition system which is equipped with object sensors and ambient sensors as described in Table 1. The sensory network of this system monitors environmental statuses (time, location, object, activity label). The stored data is processed using a specified window called an activity frame in order to check the combination of sensor information and a conditional matrix. The detail of this method is written in our previous work [19]. The main parameters that we extract in the activity recognition system are the activity labels and the duration of the performances.

### 2.2. General Architecture

**Figure 1:**
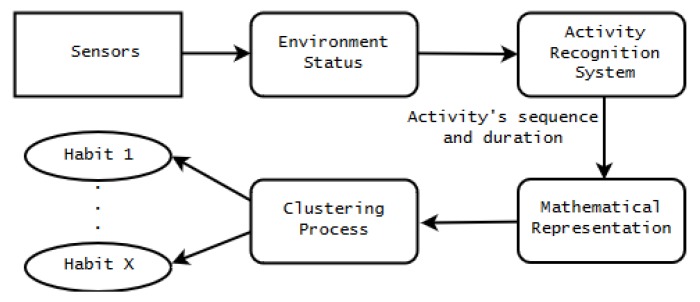
Overall architecture of the user habits assessment system. The conditional matrix and activity frame [19] were applied to extract the sequence with activity ID and duration [minutes] in this activity recognition system.

To identify the dynamic habits of users, a habit assessment system architecture based on information of a set of activities performed within a time interval is proposed, as shown in Figure 1.

First, sensor information is collected from the distributed sensors in the environments. This information (e.g., locations, activities, durations of activities) is used to understand the user’s behaviors and the state of the environment, and it recognizes a specific set of important activities in the daily living of the user. More details on the activity recognition system are available in our previous work [19]. All the observed activities within a specific time interval (e.g., morning, afternoon) are represented in a numerical format based on their occurrences, the order of identification, and the duration. For example, assume the user eats breakfast for 30 min and then watches TV for 1 h. In this case, the sequence is [1:(eating_breakfast);2:(watching_tv)], and the duration of each is [1:30_minutes;2:60_minutes]. Each activity name is associated with a specific ID allowing a precise numerical representation called the *activity ID*. Each sequence of activities, along with the durations of each activity within a specific time interval, is called a habit.

**Figure 2:**
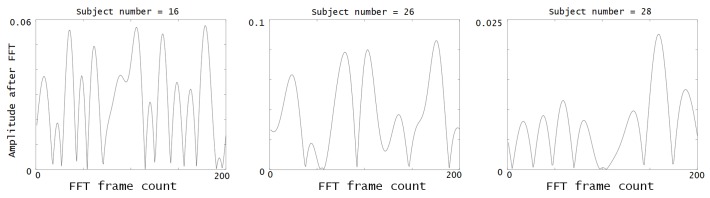
The waveform expresses a dynamic habit that includes several performed activities of user. These are examples, and the input data is in Section 4. A detailed explanation of the numerical representation is in Section 3. Note, these signals indicated positive forms because the negative part of signals inverted to positive.

**Figure 3:**
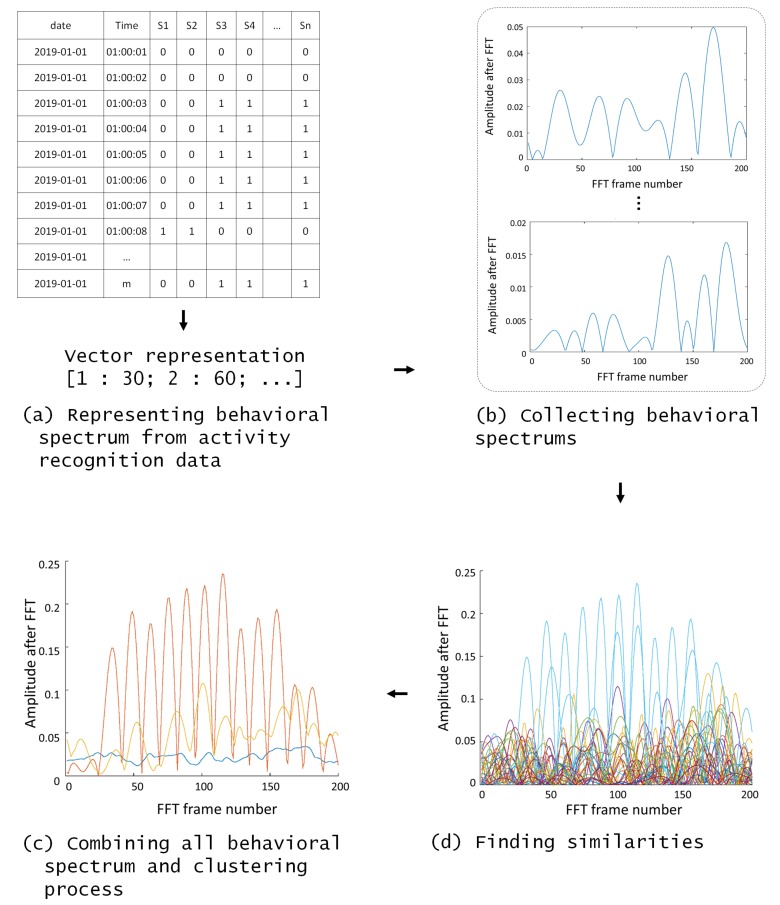
Overview of the methodology used to extract habits from the activity recognition dataset. (**a**) extracting the sequence and duration from the activity recognition system [19], and representing them as vectors; (**b**) the vectors represent a periodic signal called the behavioral spectrum. Note we coded to invert the signals of negative part to positive, in order to use only positive values when clustering the signals; (**c**) clustering all behavioral spectra; (**d**) the clustered signals are habits.

Based on these values, a numerical representation of activity is created. The numerical representation used for this work is based on the Fourier series [25], where the above-mentioned activities’ characteristics are used to create a periodic signal. The activity ID has a direct influence on the specific harmonic phase, whereas the duration affects the amplitude. The order in which an activity is performed also affects the harmonic, according to its respective number (with the exception of the first harmonic, which represents the average of the signal). Thus, the first activity in a habit is always related to the second harmonic, the second activity is associated with the third harmonic, and so on. The details on the signal creation are shown in Section 3. This signal is referred to as a *behavioral spectrum* [26], and examples are shown in the Figure 2. These signals represent the different activities that are input to find potential dynamic habits, as shown in Figure 3b.

This numerical representation is clustered into groups, each representing a category of habits with similar behaviors (See Figure 3c). For example, subject A often prays for a few minutes after taking a shower in the evening. When creating the behavior spectrum based on these factors, the numerical representative signals are collected from several days of observations. Through the clustering process, we can create a cluster representing the habit of taking a shower and praying. When an unusual behavior or a different order of activities is detected, the numerical representation of those performed behaviors does not fit in the same cluster, and it is recognized as a different habit. After clustering and analyzing each habit associated with different clusters, the similarities of sequences and the activity’s duration are found among the user’s ADLs. In Figure 3c, three different signals are clustered; these represent higher/moderate/lower frequencies in this case because the number of activities inside them is associated with the harmonic phase in the proposed method. For example, when the signal of subject A in Figure 3d is associated with the higher frequency representing a group of habits, where the user typically performs more activities, we can estimate that subject A usually performs more varied activities when compared with other subjects.

## 3. Numerical Representation of Activities

### 3.1. Behavioral Spectrum

To identify the habits of a user, two main parameters are used to mark the activities performed within a time interval. These parameters are the associated ID of the activity in a sequence and the duration of that observed activity. Each activity is associated with an ID number, and this activity ID is defined in the system beforehand in the list of activities performed. As an example, we created a scenario that contains two identifiable activities, watching TV and sleeping, whose associated activity IDs are 1 and 2, respectively. Two vectors represent the necessary parameters. One is the vector sequence containing the ID number of the activity, and the other is the vector duration that expresses the duration of each of the observed activities. Let us assume that in the given time of observation, the user slept for 10 min, and then watched TV for 60 min. Based on the activity IDs defined above, this information can be represented by the following vectors: sequence=[2,1] and duration=[10,60]. Each activity behavior is expressed as a periodic signal created using Fourier series representation. Fourier series is a powerful way of representing a periodic function as a sum of sine and cosine functions [25]. In this work, we name the output signal as *behavioral spectrum*. This representation allows the system to maintain the order of the activities, while tracking the duration of each of them. The frequency of the behavioral spectrum has a direct relationship with the activity ID, whereas its amplitude is related to the time spent in each activity. The adapted equation from the Fourier representation is as shown in Equation (1), where *j* is the number of activities arranged in the order of observation (i.e., the first activity observed is *n* = 1), $A_{n}$ is the duration in minutes of the *n*th activity, and $w_{n}$ is the ID of that same activity.

(1) $$f\left(t\right)=\sum_{n=1}^{j}A_{n}e^{iw_{n}t}$$

By using this representation method, the information regarding the sequence and the duration of each activity are mathematically expressed in the output signal. Besides the first harmonic, which represents the average value of the output signal, each harmonic in a crescent order is related to an activity according to its sequential order of observation. The phase for that specific harmonic is represented by the ID associated with that activity. The amplitude of the same harmonic is represented as the duration of the activity (in minutes). In the example described above (sleeping for 10 min and then watching TV for 60 min), the phase of the second harmonic is equal to 2 and the amplitude of second harmonic is equal to 10. The third harmonic phase is equal to 1 and the amplitude of the third harmonic is equal to 60. Both values for the phase and amplitude regarding the first harmonic are set to zero, so that no value is added to the output signal. To compute this representation concept, we used the fast Fourier transform (FFT) algorithm. Because the sequence of activities and their durations affect the waveform, we used a simple function to indicate these two parameters. Therefore, the behavioral spectrum indicates the amplitude after FFT via the FFT frame numbers (a maximum FFT frame number of 200 frames was used in this study).

**Figure 4:**
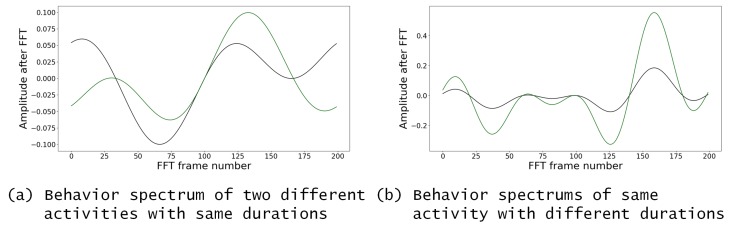
The examples of behavioral spectrum. (**a**) Two different activities performed for the same duration. Here, watching TV and sleeping were set as ID 1 and 2 respectively, and each activity was performed for a duration of 10 min. Therefore, the vector was [1:10;2:10]. The black line indicates the user who watched TV (ID 1) for 10 min. The green line shows the user who slept (ID 2) for 10 min; (**b**) is another example of a behavioral spectrum that represents sequencevector[1,2] with two different durationvectors:[10,10] and [30,30]. The amplitude of the behavioral spectrum indicates the duration of activities. The black line shows the user who watched TV before sleeping for 10 min. The green line indicates the activities being performed in the same order, but the duration is 30 min. The difference here is highlighted in the amplitude of the behavioral spectrum.

**Figure 5:**
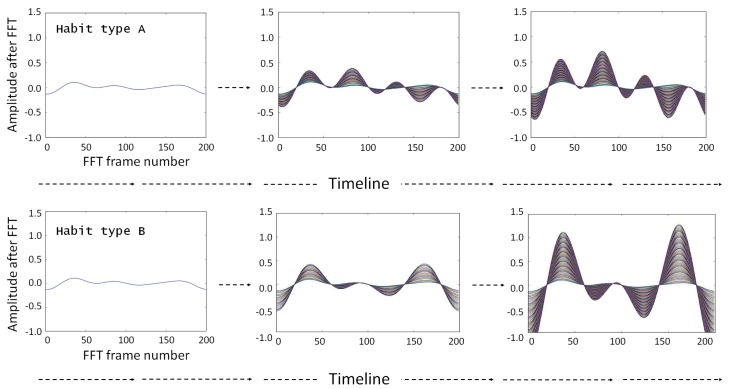
Changes of forms in the two different types of habits over a period of time.

Using the Fourier series representation, even for experiments with a different number of observed activities and duration, the behavioral spectrum always has the same size, and is defined when the signal is created. Using the data from the behavioral spectrum, this representation is capable of distinguishing the differences of a user’s activity sequences and their durations. It is important to emphasize that the similarities/differences of waveforms are present in the behavioral spectrum instead of the absolute value of FFT results before the clustering process discussed in Section 3.2. Figure 4 shows an example of two different behavioral spectra that correspond to two different activities. The waveforms of the behavioral spectra can be dissimilar depending on the activities, and Figure 4a illustrates the performances of two different types of activities having the same duration. The amplitude of the behavioral spectrum is the duration of the performance, as shown in Figure 4b. More specifically, Figure 5 illustrates the changes in the forms of the two different types of habits over the time duration, wherein $A_{n}$ represents the amplitude.

**Figure 6:**
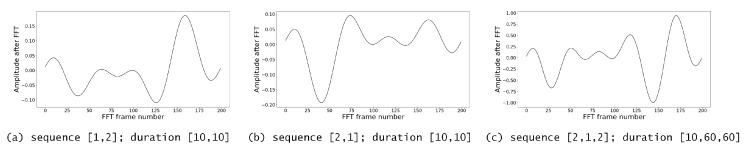
A comparison of behavioral spectra in different sequences (**a**) activity 1 finished before activity 2 started. Each performance duration was 10 min; (**b**) activity 2 finished before activity 1 started. Each performance duration was 10 min; (**c**) activity 1 performed between activity 2. The durations were 10, 60, and 60 min respectively.

Figure 6 shows examples of how the order of activities can apply to the form of a behavioral spectrum. In Figure 6a, the activity of watching TV (ID 1) was performed before the activity of sleep (ID 2), and each was performed for 10 min. Meanwhile, the user in Figure 6b slept for 10 min and then watched TV for the same duration of time.

This approach allows us to analyze a sequence of activities, thereby extracting important information regarding user behavior in the performance of a series of activities. Figure 6c shows an example of the behavioral spectrum representing one overall habit, where the user repeats one of the earlier performed activities. In this case, a user sleeps for 10 min, followed by the activity of watching TV for 60 min, and then finally sleeps for another 60 min. If the same pattern is observed in multiple behavioral spectra collected from several trials, that can be an indication of the user’s habit.

**Figure 7:**
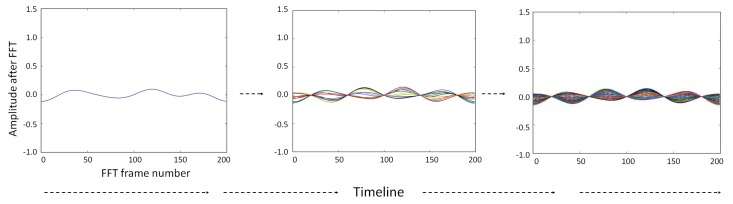
Users performed the same activities in a different order over a time period.

Figure 7 shows the case of users performing the same activities in a different order. In this case, $A_{n}$ and $w_{n}$ are constant in Equation (1).

### 3.2. Clustering Behavioral Spectrum to Find Habits

As the habit of the user is found after analyzing the patterns observed in the user activities and behaviors, it is not possible to recognize the user habits using only one instance of information. Therefore, it is necessary to analyze the user activity behavior across several days, where each pattern of behavior represents a routine or habit. This is possible after grouping several behavioral spectra in order to find similarities between the observed data. These similarities represent the habits of the user. This is carried out by a clustering process, which is a method for finding similarity groups in a given set of data [27]. It attempts to group information in a population based on similarity, but is not oriented by a specific label from output values mapped by an algorithm to data points.

As one of the objectives of this work is to compare the results of the obtained clusters, it is important to choose a clustering method that allows easy interpretation of the results. For this reason, the *k-means* method was used for clustering [27,28]. It consists of finding centroids in each data dimensionality that represent one cluster of data in that specific dimension [28]. The clustering method used in this work is a version extended from a previous work [26]. The previous version had a high chance of getting into the local minimum problem, which could lead to incorrect association between habits and some groups of sequences of activities. As explained earlier, the main purpose of the clustering method is to find similar patterns between several trials collected across different days. Each pattern found can express a different habit of the user.

Because the clustering process occurs by starting at random points automatically selected by the algorithm, it is necessary to consider several interactions in order to identify the one that would provide the best centroid position [27]. The quality of each interaction is judged according to the percentage of variance found in the index vector represented by the *k-means* algorithm. This vector correlates the observations with the corresponding clusters. Low variance indicates that the clustering interaction did not create good centroid points that represent the intended data generalization. By adding another cluster, more data would be represented by the newly added cluster, thereby spreading the representation and increasing the percentage of explained variance by the total number of clusters.

#### 3.2.1. Pre-Processing for Behavioral Spectrum Raw Data

To obtain the variability data from the behavioral spectrum, we used signal average, standard deviation, variation, auto correlation, and entropy and all parameters calculated by the pre-processing method as shown in Algorithm 1.
**Algorithm 1** Pre-processing method algorithm.1: Calculate the mean, standard deviation, variance, auto-correlation, and entropy of each raw data2: Calculate distance between each raw data and centroid all parameters.3: **if** the raw data close to centroid parameters in each, and move to the same centroid parameters.   **else** repeat step 2

Assuming that the behavioral spectrum is represented as $f=[f_{1},f_{2},\ldots ,f_{m}]$ where m is the number of points inside the behavioral spectrum, we define the above described operations in the following manner. The mean here is a sum of all points inside a behavioral spectrum divided by the vector length as shown in Equation (2).

(2) $$f_{mean}=\frac{1}{m}\left|\sum_{n=1}^{m}f_{n}\right|$$

The standard deviation is the square root of the average of the squared deviations from the mean as shown in Equation (3).

(3) $$f_{dev}=\sqrt{\frac{1}{m}{\left|\sum_{n=1}^{m}f_{n}-f_{mean}\right|}^{2}}$$

The variance is the average of the squared deviations from the mean as shown in Equation (4).

(4) $$f_{var}=\frac{1}{m}{\left|\sum_{n=1}^{m}f_{n}-f_{mean}\right|}^{2}$$

The auto correlation presents the correlation of **f** with a delayed copy of itself as a function of delay. It is represented by a correlation coefficient; furthermore, instead of the correlation between the two variables, it represents the correlation between the two values of the same variable at times $f_{n}$ and $f_{n+k}$, where k denotes the delay. This is because the auto correlation can be different for two habits with the same variance and correlation. For example, habit 1 has the sequence [1,2,3], whereas habit 2 has the sequence [3,2,1], and both of the habits have the same duration. In this case, the auto correlation differs between the habits.

Therefore, given the signal f, its auto correlation, using k=1 as delay, is defined as Equation (5)

(5) $$r_{k}=\frac{\sum_{n=1}^{m-k}\left(f_{n}-f_{mean}\right)\left(f_{n+k}-f_{mean}\right)}{\sum_{n=1}^{m}{\left(f_{n}-f_{mean}\right)}^{2}}$$

The entropy provides an appropriate measure of the randomness inside the signal **f**. The entropy *s* of a distribution **f** is calculated as Equation (6)

(6) $$s=-\sum_{n=1}^{m}\left(f_{n}*log\left(f_{n}\right)\right)$$

Finally, we define variability *v* as Equation (7). The features in *v* are average (Equation (2)), standard deviation (Equation (3)), variation (Equation (4)), auto correlation (Equation (5)), and entropy (Equation (6)); $v=[f_{mean},f_{dev},f_{var},r_{k},s]$.

(7) $$v_{i}=argmin{∥v_{n}-\hat{v_{k}}∥}^{2}$$

Variability $v_{i}$ is calculated as the distance between each variability data $v_{n}$ and centroid variability $\hat{v_{k}}$, and relates to step 3 in Algorithm 1.

#### 3.2.2. K-Mean Clustering for Behavioral Spectrum and Vvariability

As described in Section 3.2, in the pre-processing phase, five features (mean, standard deviation, variance, auto-correlation, and entropy) were calculated for the whole of the raw data (See Tables 3 and 4 in Section 5). The pre-possessed data were calculated for variability *v* in Equation (7). All of the above mentioned features were clustered separately using the k-means clustering algorithm, as described in Section 3.2. To find the optimum number of clusters, the elbow method [26] was used in both of the datasets. When the number of clusters used in the *k-means* algorithm increased, no significant increment in the percentage of explained variation was observed between the clusters and their respective indexes. The marginal point where the explained variance did not change substantially according to the number of points is represented by an apparent angle in the graph, which explains the name of the “elbow method” [29]. This method is used to calculate the proportion of explained variation $E\sigma_{u}$ (See Equation (8)) for each cluster.

The explained variance $E\sigma_{uk}$ is given by the following:

(8) $$E\sigma_{u}=argmin{∥u_{i}-\bar{u}∥}^{2}-argmin{∥u-u_{i}∥}^{2}$$

where u=[f,v] (f: behavioral spectrum, v: variability, as explained in Section 3.2)is the resulting centroid of the k-means, which represents either *f* or *v* using the number of clusters as *k*. $E\sigma_{uk}$ calculates the distance between each raw or variability data $u_{i}$ and centroid raw or variability data baru. If the variability is close to the centroid raw or variability data, move to the same centroid raw or variability data as in step 4 of Algorithm 2.
**Algorithm 2** K-means clustering algorithm for raw data and variability data.1: Initialize K randomly in the centroid points2: Repeat3: Calculate distance between each raw or variability data and centroid raw or variability data4: **if** the variability is close to centroid raw or variability data, move to the same centroid raw or variability data   **else** repeat steps 2 and 3

## 4. Data Collection and Experimental Setup

To evaluate the effectiveness of the proposed system, we collected the data from two types of users with different ages, including university students and elderly people (See Table 2). The CU smart home dataset was collected in accordance with the Chubu University ethical guidelines for research, and the HISUISUI care home dataset has been approved by the institutional review board of JAIST, which is a research partner in the CARESSES project.

**Figure 8:**
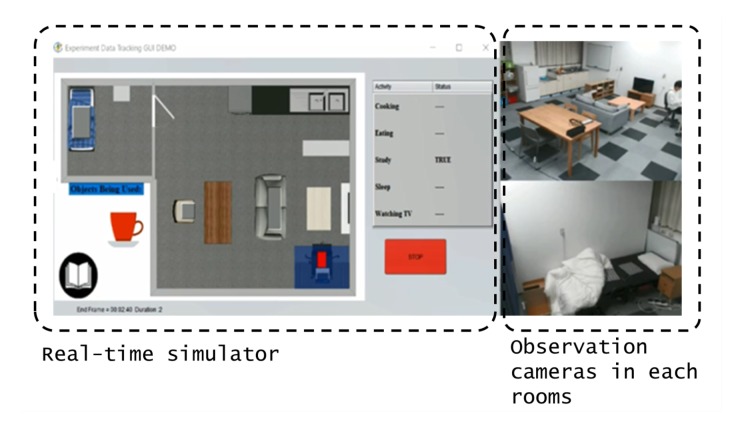
Collecting the data by the activity recognition system in CU smart home. All subjects were university students and this particular subject was studying at the time. The cup and book icons were appeared and the indicator of study changed to TRUE in the real-time simulator.

The first dataset was originally collected by the system suggested in our previous study [19,26]. The data collection environment was a facility at the University, and the area contains a kitchen, dining room, bedroom, living room, and study room. The sensors for detecting each activity (i.e., *cooking*, *eating*, *drinking*, *studying*, *sleeping*, and *watching TV*) were equipped in the furniture or objects of the aforementioned rooms, and our human activity recognition system was also installed at the facility (referred to as CU smart home in this paper) as shown in Figure 8.

This system utilized an activity frame and a conditional matrix [24]; it accurately collected all the data [24] and automatically arranged the matrix form for direct use as input data for habit estimation. Over a period of two weeks, 24 subjects (age *M*:21.71, *SD*:0.55) participated in the experiments. We note that one subject participated in the experiment four times, resulting in data being obtained from 28 subjects instead of 24. However, this does not affect the outcome of the study because the goal of this study was to represent the habit. Considering that a person could perform similar sequences of activities for similar durations every day, it is worth including such data from the same subjects. All the subjects were given the liberty to choose their activities which were performed in their desired order for a maximum of 20 min.

The second dataset was provided by the HISUISUI care home (hisui.or.jp), a care home facility for the elderly located at the Ishikawa prefecture of Japan. The daily activities of the residents over a day were annotated by a staff member of the facility. This dataset included a list of activities and durations for performing activities (25 activities in total). The number of observed residents was 12 (50% male) and had an average age of 85.25 (*SD*: 4.95). Whereas the subjects executed the selected activities within a limited time frame, the subjects in the HISUISUI care home dataset were monitored over a day, which made the HISUISUI care home dataset be more diverse in terms of activities.

The durations and sequences of activities from the both the datasets were used as parameters to express the behavioral spectrum. As mentioned in Section 3.1, we implemented the FFT algorithm to represent the behavioral spectrum. To compute this spectrum, we used the numpy.fft function from the python library (docs.scipy.org/doc/numpy/reference/routines.fft.html).

## 5. Results

### 5.1. Pre-Processing Results

Table 3 and Table 4 show the results of variability of CU smart home dataset and HISUISUI care home dataset, respectively, as described in Section 3.2. The Mean, Standard deviation, variance, auto-correlation, and entropy were calculated according Equations (2)–(6) in Section 3.2.1.

### 5.2. Explained Variance Results

**Figure 9:**
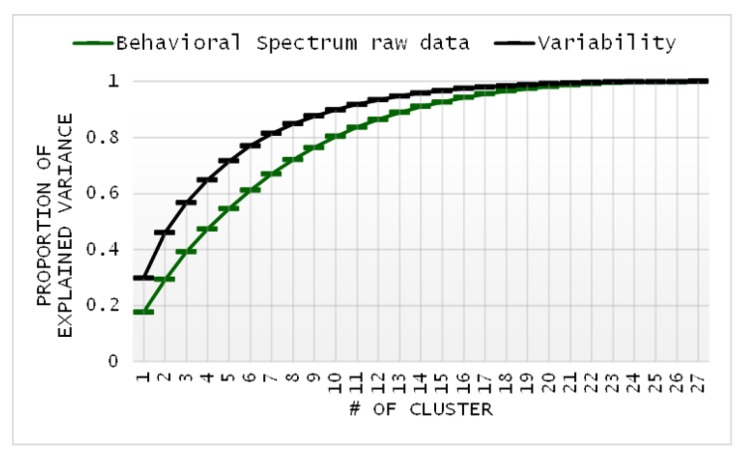
Proportion of explained variance in the data of behavioral spectrum raw data/variability by the number of clusters for the CU smart home dataset.

Figure 9 and Table 5 show the proportion of explained variance at each cluster in the CU smart home data. It shows a comparison of the proportion of explained variance between the behavioral spectrum raw data and variability, where $E\sigma_{u}$ of variability indicates a relatively higher value. $E\sigma_{u}$ is explained in Equation (8) as described in Section 3.2.2.

**Figure 10:**
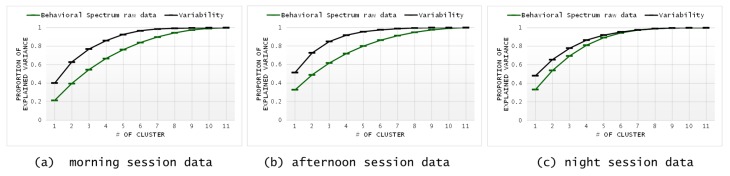
Proportion of explained variance in the data of behavioral spectrum raw data/variability by the number of clusters for the HISUISUI care home dataset.

Due to the large number of activities, the HISUISUI care home dataset was divided into three phases (morning, afternoon, and night), whereas the CU smart home data contained five different activities. In Figure 10a–c, the raw data and variability of the three-phase behavioral spectrum are presented. Table 6 indicates the proportion of explained variance at each cluster in the HISUISUI care home dataset.

## 6. Discussion and Conclusions

In this study, a methodology was presented to represent the habits of users based on the sequence and duration of their activities. To get the parameters (sequence and duration), we used the activity recognition system presented in our previous work [19,24]. The method using a conditional matrix (as explained in Section 2.1) showed a high recognition rate [24]. A method using an activity frame with a decision tree was used partially in this work and had a higher recognition rate than HMM, Naive Bayes Classifier, ALHMM, Markov Logic Network, and Neural Network methods [19]. User behavior and activity patterns can be identified through the use of behavioral spectra, which represent human activities as signals that are used as parameters in the output of the activity recognition system. As mentioned in Section 1.1, several studies focus on activity recognition but very few studies focus on habit recognition. Most of them have focused on abnormal behavior recognition by vision data [7]. To the best of our knowledge, ours is the first study to identify human habits using the assessment of signals by clustering. By comparing the percentage of explained variance obtained using both of the features to express the activity information, it was demonstrated that by using variability, it is possible to obtain a higher percentage from a lower number of clusters compared to the behavioral spectrum in its raw format. By comparing the three different periods of the day, it was shown that there is a higher chance of user performing different activities during the morning period, and this was also observed when the data annotation was analyzed. Furthermore, a relative difference was observed when comparing the explained variance by the number of clusters according to the subject ages. For example, using five clusters for elderly data, we obtained 92% to 95% of the total explained variance, while using the same number of clusters for the young population dataset (younger subjects) obtained 71% of the total explained variance. We restrain from making conclusive statements about the comparison, because the number of participants in the two groups were limited and not well balanced. However, our goal in this study was to present a human habit in a numerical manner, and the obtained results demonstrated the potential of the behavioral spectrum in representing it. Such information can be helpful for several human interaction applications, allowing access to the activity of the user as well as changes in the degree of interaction according to those activities. The knowledge regarding the association between the frequency of the observed activities and the user’s habit patterns can help in improving user experience for smart home applications and domestic robots. As a future work, we intend to expand the dataset by including the ages of the subjects and longer activity periods in order to develop a more accurate habit model.

## Figures and Tables

**Table 1:** Summary of distributed sensors.

Activity	Space	Sensor	Location of Sensor
Study	Study room	PIR(1) *, Pressure(2), RFID(1)	Ceiling, Chair, Book
Sleep	Bedroom	PIR(1), Pressure(2)	Ceiling, Bed
Cook	Kitchen	PIR(1), RFID(2)	Ceiling, Cup and
			plate
Eat	Dining room	PIR(2), Pressure(2), RFID(2)	Ceiling, Chair,
			Cup and plate
Watch TV	Living room	PIR(1), Pressure(2), Current(1)	Ceiling, Sofa, TV

* (number) is the number of sensors of a kind.

**Table 2:** The dataset description.

Place Description	# of Subject	Age	# of Activity
CU smart home	24 (all males)	21.71 ± 0.55	5 (cooking, eating, drinking, studying, sleeping, watching TV)
HISUISUI care home	12 (6 females)	85.25 ± 4.95	25 (chatting, having a breakfast, getting dressed etc.)

**Table 3:** Variability of the CU smart home dataset by pre-processing, which is used for clustering. Mean, Standard deviation, variance, auto-correlation, and entropy were calculated according Equations (2)–(6). Note that one subject participated in the experiment four times, resulting in data obtained from 28 subjects instead of 24.

Subject #	Mean	SD	Variance	Correlation	Entropy
subject01	0.02636	0.01514	0.00023	0.95544	5.11489
subject02	0.02144	0.01741	0.00030	0.96674	4.98523
subject03	0.03010	0.01850	0.00034	0.97433	5.07896
subject04	0.03526	0.02383	0.00057	0.94684	5.05074
subject05	0.02773	0.02106	0.00044	0.97946	5.01559
subject06	0.03009	0.02611	0.00068	0.97701	4.94089
subject07	0.02037	0.01644	0.00027	0.94575	4.96269
subject08	0.01769	0.01212	0.00015	0.96707	5.04646
subject09	0.02301	0.02229	0.00050	0.98585	4.87808
subject10	0.04088	0.02853	0.00081	0.86756	5.03803
subject11	0.02262	0.01523	0.00023	0.91336	5.04874
bject12	0.01466	0.01220	0.00015	0.97221	4.95487
subject13	0.01512	0.01006	0.00010	0.98118	5.07959
subject14	0.01359	0.00939	0.00009	0.94410	5.04959
subject15	0.00482	0.00446	0.00002	0.98076	4.90523
subject16	0.02721	0.01593	0.00025	0.94297	5.10057
subject17	0.02326	0.02073	0.00043	0.98243	4.92686
subject18	0.03803	0.02757	0.00076	0.96903	5.00913
subject19	0.02895	0.02101	0.00044	0.93282	5.03711
subject20	0.07096	0.04891	0.00239	0.95730	5.04015
subject21	0.02428	0.01930	0.00037	0.98511	4.98775
subject22	0.01978	0.01450	0.00021	0.98122	5.01609
subject23	0.01885	0.01410	0.00020	0.94885	5.00614
subject24	0.03259	0.02871	0.00082	0.97857	4.94494
subject25	0.00666	0.00599	0.00004	0.97930	4.92529
subject26	0.03703	0.02388	0.00057	0.98390	5.07271
subject27	0.09684	0.06781	0.00460	0.92677	5.02007
subject28	0.00707	0.00524	0.00003	0.98309	5.01965

**Table 4:** Variability of the HISUISUI care home dataset by pre-processing, which is used for clustering. Mean, Standard deviation, variance, auto-correlation, and entropy were calculated according Equations (2)–(6).

Subject #	Mean	SD	Variance	Correlation	Entropy
subject A	0.02636	0.01514	0.00023	0.95544	5.11489
subject B	0.02144	0.01741	0.00030	0.96674	4.98523
subject C	0.03010	0.01850	0.00034	0.97433	5.07896
subject D	0.03526	0.02383	0.00057	0.94684	5.05074
subject E	0.02773	0.02106	0.00044	0.97946	5.01559
subject F	0.03009	0.02611	0.00068	0.97701	4.94089
subject G	0.02037	0.01644	0.00027	0.94575	4.96269
subject H	0.01769	0.01212	0.00015	0.96707	5.04646
subject I	0.02301	0.02229	0.00050	0.98585	4.87808
subject J	0.04088	0.02853	0.00081	0.86756	5.03803
subject K	0.02262	0.01523	0.00023	0.91336	5.04874
subject L	0.01466	0.01220	0.00015	0.97221	4.95487

**Table 5:** Explained variance by the number of clusters in the CU smart home data. Note that one subject participated in the experiment 4 times, resulting in data with 28 subjects instead of 24.

# of Cluster	Behavioral Spectrum Raw Data	Variability
1	0.175929	0.298417
2	0.292888	0.460526
3	0.391659	0.567007
4	0.473769	0.648469
5	0.546723	0.71612
6	0.611369	0.770613
7	0.669793	0.814549
8	0.720086	0.848649
9	0.764429	0.876502
10	0.803406	0.898756
11	0.836891	0.918295
12	0.864667	0.934148

**Table 6:** Proportion of explained variance in the data by the number of clusters for activity information (all periods of the day).

# of Clusters	Morning		Afternoon		Night	
	BS Raw	Var	BS Raw	Var	BS Raw	Var
1	0.215221	0.402779	0.328127	0.515181	0.33304	0.482183
2	0.39694	0.629449	0.490054	0.726513	0.539277	0.655905
3	0.54675	0.769499	0.617247	0.848972	0.695666	0.77942
4	0.667462	0.860869	0.719558	0.917877	0.811862	0.866686
5	0.763218	0.926923	0.800429	0.95498	0.893755	0.921042
6	0.839136	0.966721	0.863255	0.975527	0.945263	0.954697
7	0.899722	0.985933	0.912124	0.988503	0.975252	0.976818
8	0.944545	0.993891	0.948865	0.995058	0.990342	0.990635
9	0.975126	0.998269	0.975361	0.998653	0.997732	0.997056
10	0.992345	0.999517	0.991979	0.999627	1	1
